# Characterisation and Modelling of PLA Filaments and Evolution with Time

**DOI:** 10.3390/polym13172899

**Published:** 2021-08-28

**Authors:** Jaime Orellana Barrasa, Ana Ferrández-Montero, Begoña Ferrari, José Ygnacio Pastor

**Affiliations:** 1 Departamento de Ciencia de Materiales-CIME, Universidad Politécnica de Madrid, 28040 Madrid, Spain; jy.pastor@upm.es; 2 Instituto de Cerámicay Vidrio (CSIC), Campus de Cantoblanco, 28049 Madrid, Spain; ana.ferrandez-montero@cyu.fr (A.F.-M.); bferrari@icv.csic.es (B.F.); 3 Laboratory of Physicochemistry of Polymers and Interfaces (LPPI), CY Cergy Paris University, Neuville-sur-Oise, 95031 Cergy, France

**Keywords:** PLA, FDM, extrusion, thermo-mechanical properties, ageing, strain rate, printing temperature, analytical models, logistic model

## Abstract

The properties of polylactic acid (PLA) filaments have not yet been analysed in detail, and they are strongly affected by the extrusion process used in some additive manufacturing systems. Here we present the mechanical, thermal, physical, and fractographical properties of an extruded filament (not the bulk material or scaffolds), the basic building block of any PLA structure printed via material extrusion. This research aims to create a reference point for the modelisation of additively manufactured structures via extrusion processes, as the main building block is characterised in detail for a deep understanding. Furthermore, we investigated the natural ageing (up to one year), the effect of the printing (extruding) temperature (180 and 190 °C), and the effect of the crosshead speed during the tensile tests (10^−1^ to 10^2^ mm/min) to provide a deeper analysis of the material. The results showed that the material extruded at 190 °C performed better than the material extruded at 180 °C. However, after one hundred days of natural ageing, both materials behaved similarly. This was related to the flow-induced molecular orientation during the extrusion. The crosshead rate produced a logarithmic increase of the mechanical properties, consistent with the Eyring model. Additionally, the ageing produced significant changes in both the elastic modulus and the yield strength: from 2.4 GPa and 40 MPa, in one-day-aged samples, up to 4 GPa and 62 MPa once entirely aged. Finally, it was observed that the glass transition and the enthalpic relaxation increased with ageing, agreeing with the Kohlraushch–William–Watts model.

## 1. Introduction

Among synthetic biodegradable plastics, poly(lactic acid) (PLA) is an amorphous and compostable polymer derived from renewable sources [1,2]. Agricultural feedstocks, such as corn, starch, and sugar beet, are currently used as raw materials for sustainable production. Other alternatives, including biomass and low-value by-product wastes, are also considered today to overcome the potential competitive issues with human and livestock food supply chains. PLA is considered a promising material due to its complete biodegradability—although the meaning and value of this property are highly dependent on the standard used [3,4,5,6,7,8]—thermoplasticity, high transparency, moderate water resistance, and commercial availability. These properties have facilitated PLA’s application in products for human consumption; for example, in food packaging [9] and medical science [10], including manufacturing of tissues and organs [11], biodegradable scaffolds [12], and biodegradable endoprostheses [13]. PLA degrades in aqueous media to form monomers that quickly metabolise water and carbon dioxide as natural compounds, closing its eco-friendly life cycle. Unlike other biodegradable polymers such as PGA (polyglycolic acid), its degradation rate is slow enough to produce a rejection in the body with biodegradation products [14] or other biocompatible polymers which do not exhibit these biodegradable mechanisms, like PMMA [15].

These are significant arguments supporting the identification of PLA as one of the most commonly used and commercially available thermoplastic vehicles/matrixes in additive manufacturing (AM) of composites and nanocomposites by material extrusion [1,9,16,17,18]. Although 3D printed PLA—which is extruded PLA that is deposited layer by layer at specific locations—exhibits a high dimensional accuracy due to its low thermal expansion coefficient, several properties still need to be improved, especially in applications where PLA is intended to be used as a substitution for existing thermoplastics, such as acrylonitrile butadiene styrene (ABS), polyvinyl alcohol (PVA), polycaprolactone (PCL), and polyamide (nylon). The rough surface of 3D printed parts and the brittleness of PLA can prevent its use in applications where toughness and impact resistance are critical. Moreover, PLA is biodegradable, degraded by the ambient humidity [19,20], and has low barrier properties, resulting in unpredictable performance if the polymer is exposed to uncontrollable temperature and humidity conditions. In this sense, ageing studies that take a comprehensive perspective will be helpful.

Numerous authors have studied the bulk material and three-dimensional (3D) structures of PLA printed via material thermal extrusion [21,22]—for simplicity, just extrusion—considering variables such as printing speed [23], printing temperature [24], printing height between layers [25,26], printing bed temperature [27], post-printing treatments [28], and pigmentations [29] and other additives [30]. However, very little information can be found in the literature about the explicit characterisation of single PLA filaments, which are one-dimensional (1D) structures and the basic building blocks of any 3D printed structure after the extrusion process.

This paper provides information on 1D PLA structures, specifically the single filaments (not the bulk material or scaffold structures), manufactured via the extrusion process with a homemade 3D printer, and analyses the natural ageing and thermal, physical, mechanical, and fractographical properties. Furthermore, the importance of the loading speed during mechanical characterisation is explored. Studying the deformation micro-mechanisms of amorphous polymers makes it possible to understand the change from ductile to fragile behaviour with increasing testing speed [31]. The influence of the printing temperature on the ageing and mechanical properties is also analysed. Note that, throughout the text, “printing” and “extrusion” are used as synonyms.

Several effects take place during the extrusion of PLA, such as flow-induced molecular orientation [32,33,34] and flow-induced crystallisation [35]; during the ageing, such as hydrolysis, photo-oxidation, thermal decomposition, and natural weathering, which are highly dependent on the ageing conditions (ambient humidity, temperature, pH, and exposure to ultraviolet light) [36,37,38,39,40,41,42,43,44,45]; during the thermal tests, such as the crystallisation in different crystalline phases [46,47]; and during the mechanical tests, such as the change of mechanical properties with the strain rate due to the viscoelastic behaviour, which is directly dependant on the amorphous phase of the PLA [48], among many possible examples. Moreover, most of these effects are interrelated and thus should be carefully analysed [49,50,51].

Some of these effects were studied here and the results obtained were adjusted to the Eyring model [52], the Kohlraushch–William–Watts (KWW) model [53], and to a logistic fitting proposed here, all of which made it possible to obtain precise predictive equations on the behaviour of the analysed material with the crosshead speed and with ageing. Thus, it is intended for this work to serve as a support for the simulation of printed structures starting from the properties of the fundamental elements, the 1D structures printed via material extrusion, and to open new possibilities in the simulation and optimisation of 3D printed structures [54,55,56,57].

## 2. Materials and Methods

**Figure 1:**
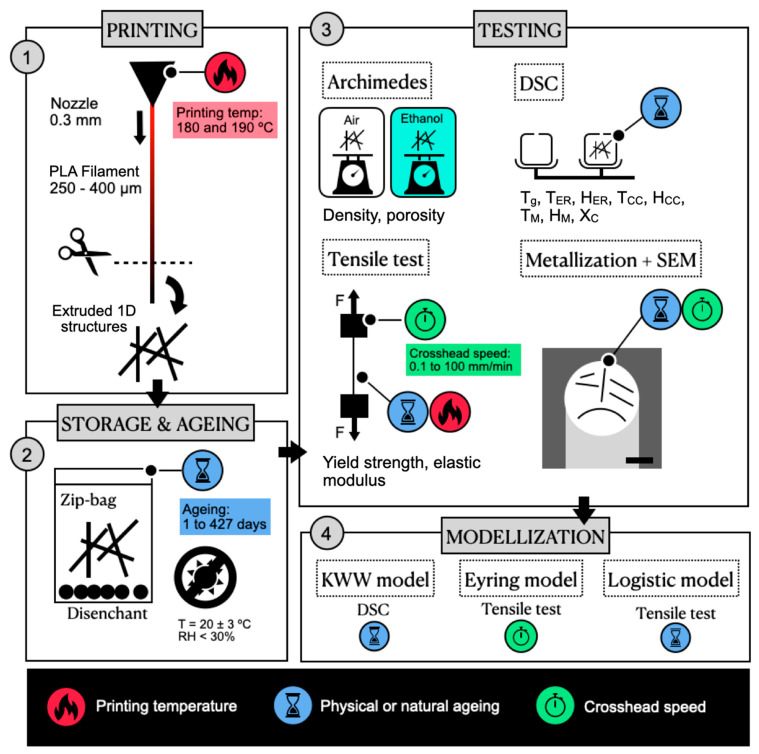
Summary of the Materials and Methods section. The material was extruded, stored, and tested, analysing the influence of the printing temperature, ageing time, and crosshead speed. The thermal and mechanical properties were modelled following the Eyring model, the Kohlraushch–William–Watts (KWW) model, and a logistic model.

A summary of the Materials and Methods section is provided in Figure 1.

### 2.1. Material Preparation and Storage

The material studied was the Ingeo biopolymer 2003D from the material supplier NatureWorks, a high molecular weight PLA (Mw = 182.000 g/mol) with 96% l-lactide and 4% d-lactide derived from bioresources and designed for food packaging applications. This material has a specific density of 1240 kg/m^3^, a yield strength of 53 MPa, and an elastic modulus of 3.5 GPa. The Mw was considered stable through all the experimentation, as low humidity values prevent the degradation of the PLA [20]. To ensure these low humidity conditions, the PLA was stored inside zip-bags with silica desiccant inside, preventing the degradation of the PLA during the ageing.

The material was printed at room conditions via an extrusion process in a homemade 3D printer with a 0.3 mm nozzle at the Institute of Glass and Ceramic (ICV, CSIC, Madrid, Spain). The extrusion process was undertaken vertically to ensure a rounded shape on the filament, as schematised in Figure 1. The printing temperatures were 180 ± 1 °C, 190 ± 1 °C, and 200 ± 1 °C.

Extruded filaments were aged in a room without direct solar light at 20 ± 3 °C inside PET zip-bags, adding silica desiccant inside the bags. The zip-bags provided great control over the humidity, maintaining a stable silica colour. Samples were aged from 1 day up to 426 days (14 months).

The morphology of the extruded filaments was studied with a Nikon Profile Projector V-12B, within a resolution of ±1 µm. The variability of the diameter through the length of the filament was used to measure the quality of the extruded material. The sections of the filaments were considered circular. Only those filaments with the right consistency in the diameter were characterised, as this was considered essential for the mechanical characterisation.

### 2.2. Physical Characterisation

The Archimedes test was used to measure the density and calculate the closed porosity. For this, a METTLER Toledo balance coupled to an LC-Density was used. The sample was weighted outside and inside ethanol at 25 °C.

The closed porosity was calculated using the material’s theoretical density (1240 kg/m^3^) and the measured density of the material.

### 2.3. Thermal Analysis

Thermal analysis was performed with a differential scanning calorimeter (DSC Mettler Toledo 822e). The temperature was raised from 30 to 220 °C at a heating rate of 10 °C/min. The weight was 5 to 10 mg, and the filaments were cut into pieces of 3–4 mm in length and placed inside a 40 µL aluminium crucible. Before the tests, the DSC apparatus was calibrated with the Indium standard.

**Figure 2:**
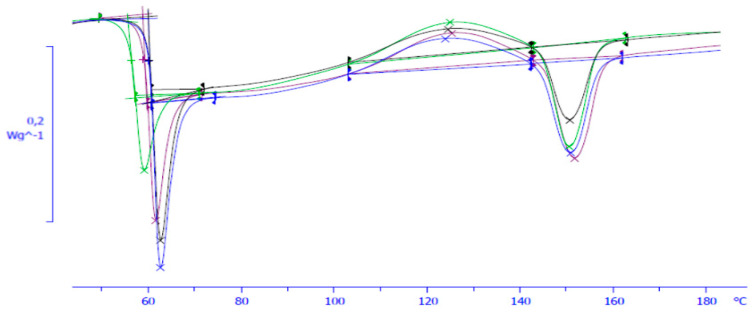
An example of the DSC results obtained from the PLA at different ageing times. Green: 14 days ageing; purple: 28 days ageing; black: 84 days ageing; blue: 140 days ageing.

The glass transition (Tg) temperature was obtained from the first endothermic second-order transition found in the DSC diagram. The value was directly calculated with STARe software. Enthalpic relaxation was calculated as the area on the endothermic curve associated with the Tg. Crystallinity was calculated by measuring the cold crystallisation and melting enthalpies. The melting point was calculated from the last endothermal curve of the DSC. The peak maximum of the melting endotherm was used for setting the Tm of the material. Figure 2 shows some of the DSC results obtained for the material studied at different ageing times.

### 2.4. Mechanical Characterisation and Fractography

**Figure 3:**
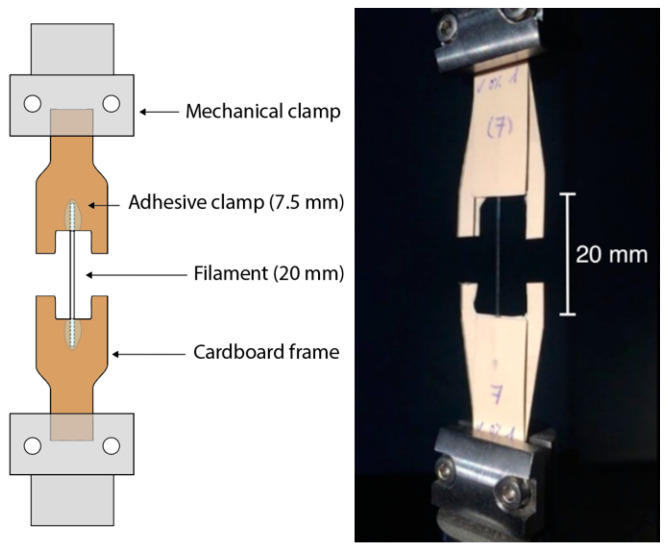
Tensile test clamp-scheme used to avoid inducing mechanical damage with the mechanical clamps.

The uniaxial tensile tests were performed according to the ISO 527-3: 2019 with the universal testing machine INSTRON 5866 using a 1 kN load cell. PLA filaments 35 mm in length were tested. The endings comprising 7.5 mm of the filaments were glued for each size to a cardboard frame (10 mm width and 500 mm thickness) to avoid inducing mechanical damage on the filament with the clamps, leaving 20 mm of the filament length for testing. The mechanical clamps were used to hold the cardboard, as shown in Figure 3. Samples were tested at crosshead speeds of 0.125, 0.5, 1.0, 5.0, 10.0, 50.0, and 100 mm/min (strain rates of 6.25 × 10^−3^, 2.5 × 10^−2^, 5 × 10^−2^, 2.5 × 10^−1^, 5 × 10^−1^, 2.5, and 5 min^−1^) at room conditions. The samples subjected to the study of the influence of the crosshead speed were aged for 10, 125, and 426 days. A minimum of six samples were tested for each condition.

A reference condition was chosen against which the different variations of each of the parameters studied were set: 190 °C for the printing temperature, 1 mm/min for the crosshead speed (5 × 10^−2^ min^−1^ for the strain rate), and a minimum of 60 days of natural ageing. This reference was set through experimentation, finding the optimum and most stable conditions according to the applicable norm.

The yield strength was calculated by dividing the maximum load during the tensile test over the minimum diameter found on the 20 mm filament before the test. It was challenging to identify the primary fracture surface of the failure specimen, but the minimum diameter was the most probable place to break the sample. The diameter was measured with a Nikon Profile Projector V-12B profilometer. The elastic modulus was calculated with the average area by computing it at five different zones (0, 5, 10, 15, and 20 mm).

The micrographs of the tested samples were taken with an AURIGA FESEM ZEISS. To avoid PLA electrical charge during FESEM observation, the specimens were metallised with a 20 nm thick carbon coating in a carbon metalliser from LEIKA. In addition, all samples were placed over brass plates and attached to them with a conductive carbon film.

### 2.5. Analytical Models and Parametrical Analysis

#### 2.5.1. Analytical Model of the Strain Rate

In the literature, different models can be found that describe the evolution of the mechanical properties of a polymer with the strain rate, like the power-law-based model [58], the original Eyring model [52], the Ree and Eyring model [59], and the cooperative model [60], among others. All of them include a logarithmic relation between the mechanical properties and the strain rate. The advanced models describe bilinear behaviour in which the polymer properties increase drastically at high strain rates. The transition strain rate usually occurs at 6000 min^−1^ [61]. As the strain rates studied here were below the transition strain rate of 6000 min^−1^, the original Eyring model was used, which complied with our test conditions [52]:

(1) $$\sigma_{y}=\sigma_{0}+\frac{kT}{V_{0}}\ln\left(\frac{\dot{\varepsilon }}{{\dot{\varepsilon }}_{0}}\right)$$

where $\sigma_{y}$ is the yield stress, $\sigma_{0}$ is the yield stress at ${\dot{\varepsilon }}_{0}$, k is the Boltzmann constant, T is the absolute temperature, $V_{0}$ is the activation volume, $\dot{\varepsilon }$ is the strain rate, and ${\dot{\varepsilon }}_{0}$ is the reference strain rate, which was 5 × 10^−2^ min^−1^ for this study.

R squared (R^2^) was used to measure how good the analytical model was.

#### 2.5.2. Analytical Model of the Ageing

There are several models to describe the ageing evolution of the material. We used the Kohlraushch–William–Watts (KWW) model [53], in the form of Equation (2), which provided an excellent fitting for the evolution of the glass transition and the enthalpic relaxation.

(2) $$\Delta \Phi \left(t\right)=\Phi \left(t\right)-\Phi \left(t-1\right)=exp\left(-{\left(\frac{t}{\tau_{0}}\right)}^{\beta }\right)$$

where ΔΦ(t) is the property modelled with the ageing (e.g., glass transition, enthalpic relaxation temperature, enthalpic relaxation enthalpy); β is the stretching exponent or relaxation distribution parameter, which is considered constant over time; t is the time in days; and $\tau_{0}$ is the enthalpy relaxation time constant.

To calculate the absolute evolution of the property Φ(t), and not its step change, the step change of each day, ΔΦ(t), was summed to the property at one day of ageing, $\Phi_{0}$, as follows:

(3) $$\Phi \left(t\right)=\Phi_{0}+\overset{n}{\underset{i=1}{\sum }}\Delta \Phi \left(t_{i}\right)={\;\Phi }_{0}+\overset{n}{\underset{i=1}{\sum }}exp\left(-{\left(\frac{t_{i}}{\tau_{0}}\right)}^{\beta }\right)$$

where *n* is the number of ageing days at which the value of the property is calculated. Values for β and $\tau_{0}$ were expected to be between 0 and 1 and between a few hours and several days, respectively [62,63,64].

For the parametrical analysis of the evolution of the mechanical properties with ageing, it was found that a logistic fitting provided more remarkable agreement than the KWW using the same number of parameters. The expression used for the logistic fitting was the following:

(4) $$S\left(t\right)=\frac{S_{\infty }\;}{1+{\;\mathrm{Ae}}^{-\mathrm{Bt}}}=\frac{\left(1+A\right)S_{0}\;}{1+{\;\mathrm{Ae}}^{-\mathrm{Bt}}}$$

where:

- $S_{\infty }$ is the property at an infinite time of ageing that corresponds to the stabilised value (steady state);
- An (adimensional) ageing potential describes the gap between the property at zero days of ageing and infinite days of ageing. A value of zero for A means that there is no gap, and thus $S_{o}$ = $S_{\infty }$.
- B (1/time) is the ageing rate or driving force, which describes how fast the material will evolve towards the steady state.

There is a parameter that can be helpful. However, it is not an independent variable: $S_{o}$, the value at 0 days of ageing. The importance of this parameter is that $S_{\infty }=\left(1+A\right)\cdot S_{o}$ and we can predict a value for the steady property $S_{\infty }$.

**Figure 4:**
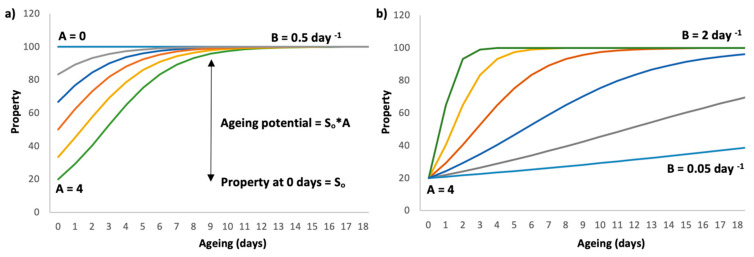
Representation of the logistic fitting for: (**a**) different ageing potentials, described by A and (**b**) different ageing rates, described with B. Note that the steady state, $S_{\infty }$, was chosen to have a constant value of 100 for all the graphs and that the time required to reach it changes with B, while $S_{o}$ varies with the value of A.

Several curves for these parameters are represented in Figure 4. Lower values of A imply that the extruded material properties are closer to those in the steady state. Lower values of B mean that the time needed to reach the steady state is greater.

**Figure 5:**
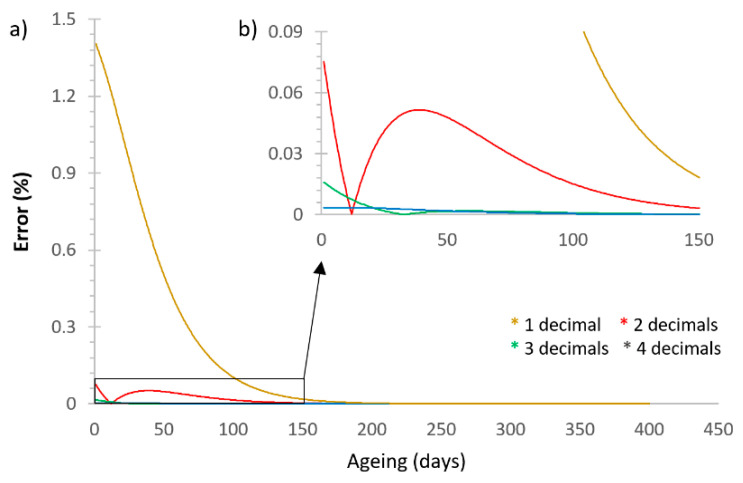
(**a**) Example of the error produced by decreasing the significant figures in A and B, compared with the results obtained for A and B with eight figures. Three significant figures were chosen as this provided an error smaller than 0.02%. (**b**) Detail of the curves.

These three parameters ($S_{\infty }$, A, and B) were analytically determined by the best fit to the experimental results. Three significant figures were used for the ageing potential (A) and the driving force (B) as they provided an error smaller than 0.02% compared to the fitting with eight significant digits, as shown in Figure 5.

## 3. Results and Discussion

### 3.1. Morphology

**Figure 6:**
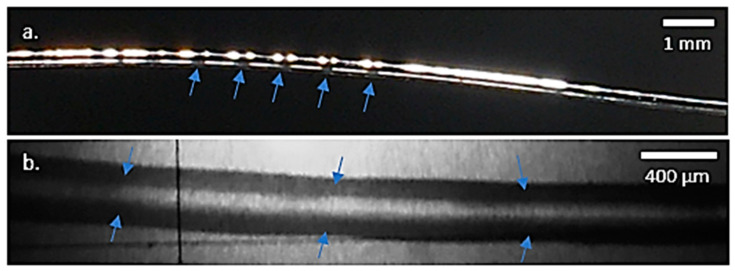
Optical microscope images: (**a**) wavy morphology on a filament extruded at 180 °C; (**b**) detailed wavy morphology, with valleys indicated with arrows.

The samples extruded at 180 and 190 °C had diameters between 250 and 400 µm, varying smoothly along the filament length. Some specimens, mainly those extruded at 180 °C, showed a wavy morphology. The diameters differed from the maximum and minimum values by 20–30% every 1 to 2 mm, as indicated in Figure 6 with arrows. However, the experimental results indicated that this phenomenon did not influence the mechanical properties. The samples extruded at 200 °C using the processing method in which the material was extruded in the air without any support to ensure the roundness of the samples were not suitable for mechanical characterisation due to highly varying diameters, and they were discarded in this study. However, the printing temperatures used for the material extrusion of PLA in additive manufacturing structures can be as high as 240 °C [28,65,66].

### 3.2. Physical Properties

The measured density of the PLA after 60 days of ageing was 1238 ± 1 kg/m^3^ for our reference material.

With the PLA density of 1240 kg/m^3^ provided by the material supplier, 0.13% closed porosity was obtained. This was in accordance with the fractographies in which no porosity was observed.

The degree of crystallinity calculated from the DSC results was almost 0%. The crystallinity values were close to 0%, as expected from our previous experience [67].

### 3.3. Thermal Properties

A summary of the thermal properties is shown in Table 1.

The ageing modified some of the thermal properties; others remained stable. The properties that increased were the glass transition (T_g_) and the enthalpic relaxation (both the temperature peak and enthalpy). In addition, the amorphous phase here became more stable with ageing. This was because the stability of the polymer was not obtained during the cooling period after the filament production but slowly with time.

The properties that remained stable were those that vary according to the crystalline phase or chemical nature of the polymer. They were not meaningfully changed with the natural ageing studied, indicating a stable molecular weight value, as expected from [20]. These properties were the melting enthalpy (ΔH_m_), the melting temperature (T_m_), the cold crystallisation enthalpy (ΔH_CC_), and the cold crystallisation temperature (T_CC_). T_CC_ and T_m_ maintained values of 125 ± 1 °C and 151 ± 1 °C, respectively.

**Figure 7:**
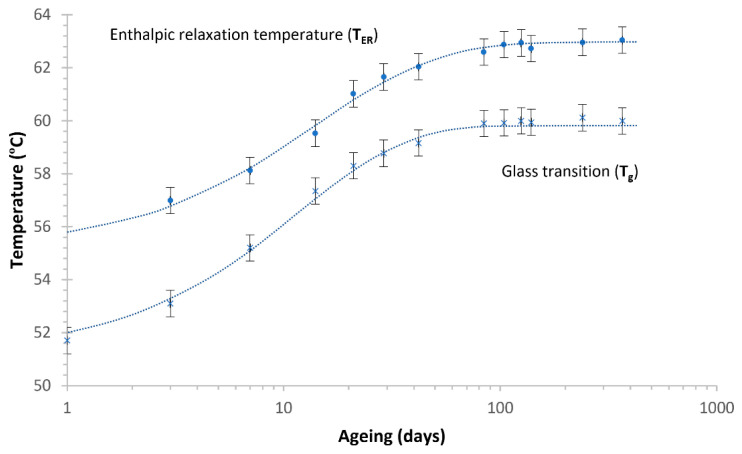
The glass transition and enthalpic relaxation temperatures at different ageing times with the corresponding KWW fitting equations (dashed lines).

The T_g_ rose from 51 °C at 1 day of ageing to almost 60 °C after 84 days. After that time, the value reached a stable region and showed no variations. The parameters obtained for the Kohlraushch–William–Watts (KWW) model were β = 0.77 and $\tau_{0}$ = 8.05 days, which showed excellent agreement with the experimental data for the evolution of T_g_ with age, as shown in Figure 7. Regarding the enthalpic relaxation temperature, the parameters obtained for the KWW model were β = 0.578 and $\tau_{0}$ = 5.25 days, and the stabilisation time could be set again at 84 days.

**Figure 8:**
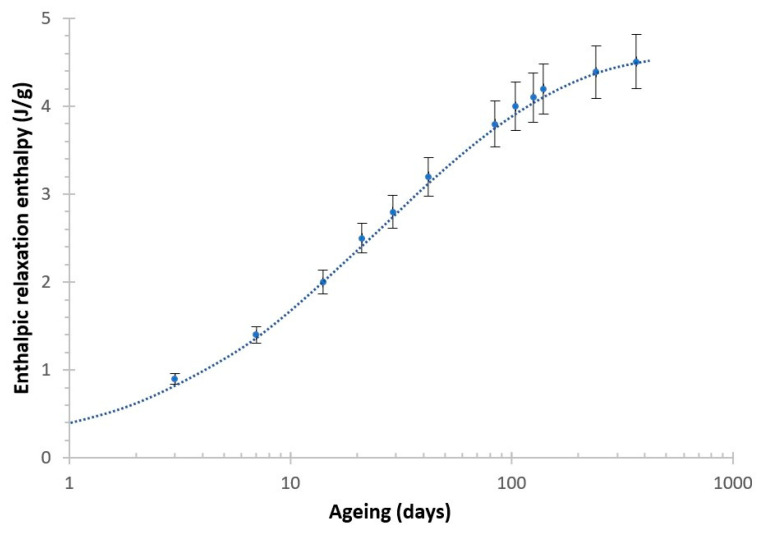
Enthalpic relaxation enthalpy at different ageing times with the corresponding KWW fitting equation (dashed line).

The enthalpic relaxation enthalpy (ΔH_ER_) increased even up to 366 days (1 year). The parameters obtained with the KWW model were β = 0.321 and $\tau_{0}$ = 0.705 days for the enthalpic relaxation enthalpy, which showed remarkable agreement with the enthalpic relaxation data with age, shown in Figure 8.

Following the thermal analysis of the extruded PLA filaments, it could be concluded that the ageing of the PLA modified the glass transition and the enthalpic relaxation, which are associated with the amorphous phase of the polymer, but not the properties related to the crystalline phase. The experimental variations of these parameters collected in Table 2 (Tg, TER, and ΔHER) fit well with the KWW model, and thus it is proven that the KWW can be used to predict these PLA properties. The data plotted in Figure 7 show that the PLA extruded aged during the first 100 days and then remained stable. Thermal and mechanical properties are related. Consequently, the evolution of the thermal properties due to ageing implies that the mechanical properties are affected by the ageing, and thus they should be studied.

Curiously, the thermal properties related to the crystalline phase of the polymer, such as the cold crystallisation and the melting temperature, were not modified. These parameters affect the printing process of the PLA filaments. Thus, the fact that they were not affected by the ageing time indicates that the same printing conditions can be used to reprocess the filaments with material held for at least one year in the storage conditions here described.

### 3.4. Mechanical Behaviour

A summary of the mechanical properties is shown in Table 2.

#### 3.4.1. Influence of the Crosshead Speed (or Strain Rate)

The yield strength and the elastic modulus increased with the crosshead speed, as shown in Table 2, which was coherent with the theory about the mechanical behaviour of polymers [52,58,59,60,61,68].

The influence of the crosshead speed on the mechanical properties of the PLA can be addressed with regard to the micro-mechanisms of deformation on polymers. When a force is applied, the polymer chains rearrange into more stable configurations to retain the excess energy applied to the material by the induced stresses. The cold-drawing phenomenon (or the propagation of the necking through the filament length) happened less often with the increase of the ageing time and the rise in the strain rate, as cold drawing requires the movement and relocation of the polymer chains. This relocation requires time, and the faster the crosshead speed, the less time the polymer chains have to rearrange into other configurations. The more the relocation was impeded, the more probable it was that a fracture of the PLA would occur, and the material would behave as a more rigid and brittle material, with less plastic deformation.

The mechanical properties were approximated with a logarithmic function, obtaining the following equations:

At 10 days of natural ageing:

(5) $$\sigma_{y}\left(\mathrm{MPa}\right)=4.94\ln\left(\dot{\varepsilon }\right)+60.0;\;R^{2}=0.975$$

(6) $$E\left(\mathrm{GPa}\right)=0.223\ln\left(\dot{\varepsilon }\right)+3.86;\;R^{2}=0.986$$

At 125 days of natural ageing:

(7) $$\sigma_{y}\left(\mathrm{MPa}\right)=3.56\ln\left(\dot{\varepsilon }\right)+69.7;\;R^{2}=0.999$$

(8) $$E\left(\mathrm{GPa}\right)=0.0925\ln\left(\dot{\varepsilon }\right)+3.68;\;R^{2}=0.936$$

**Figure 9:**
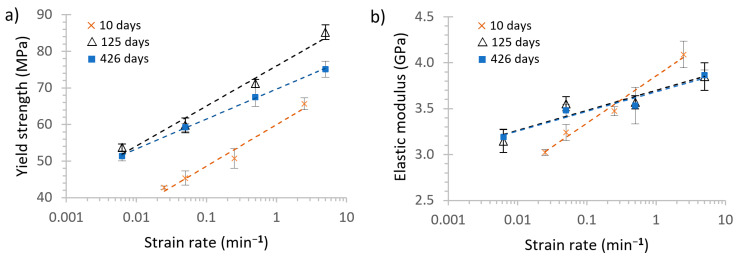
Strain rate in semi-logarithmic scale for PLA naturally aged for 10, 125, and 426 days vs. (**a**) yield strength and (**b**) elastic modulus. Logarithmic fittings are shown with dashed lines.

At 426 days of natural ageing:

(9) $$\sigma_{y}\left(\mathrm{MPa}\right)=4.75\ln\left(\dot{\varepsilon }\right)+61.7;\;R^{2}=0.979$$

(10) $$E\left(\mathrm{GPa}\right)=0.0878\ln\left(\dot{\varepsilon }\right)+3.65;\;R^{2}=0.986$$

where $\sigma_{y}$ is the yield strength in MPa, E is the elastic modulus in GPa, $\dot{\varepsilon }$ is the strain rate in min^−1^, and the independent parameter is the value of the property calculated ($\sigma_{y}$ or E) at a strain rate of 1 min^−1^. In Figure 9, the mechanical properties versus the strain rate and the analytical Equations (5)–(10) are represented. This logarithmic fitting provided an excellent approximation with regard to the R squared (R^2^) values obtained.

A limit of 100 days of ageing could again be established, which was coherent with the thermal data obtained. Several authors have stated that the ageing of PLA decreases the values of its properties [19]. However, in this study, we prevented the degradation of the PLA by decreasing the ambient humidity by storing the samples inside zip-bags with silica desiccant inside [20]. Once the material had completed aged (steady state), the strain rate impact was minor. According to the Ree–Eyring yield model [68], the yield strength and elastic modulus of a polymer depend on the rotational movements allowed to the polymer chains, and this has also been established experimentally [69]. They are also related to the internal viscosity of the polymer, or the ease with which the polymer chains flow, as the lower the mobility of the chain is, the more complex the flow and the higher the internal viscosity [70].

Moreover, this internal viscosity is related to the loss modulus. It has recently been observed that the loss modulus of the PLA decreases with ageing as the chains on the amorphous phase reach more stable locations [20]. This study obtained this relation between the more stable locations of the polymer chains and the mechanical properties by measuring enthalpic relaxation. Our results for the PLA are coherent with the well-established polymer models described previously. This proves that it is feasible to predict the mechanical properties of PLA at different low strain rates by using the logarithmic relation in the Eyring model.

#### 3.4.2. Influence of the Ageing and Printing Temperature

Regarding the data in Table 2, it can be observed that increasing the ageing of the sample also rapidly increased the mechanical properties up to a threshold of 100 days, after which the mechanical properties of the PLA slowly stabilised. The cold-drawing phenomenon happened less often with the increase of the ageing time.

The equations that were obtained to better describe the mechanical properties of the PLA with the ageing were the following:

Extruded at 180 °C:

(11) $$\sigma_{y}\left(\mathrm{MPa}\right)=\frac{58.8}{1+0.777e^{-0.0631t}}$$

(12) $$E\;\left(\mathrm{GPa}\right)=\frac{3.62}{1+0.678e^{-0.0759t}}$$

**Figure 10:**
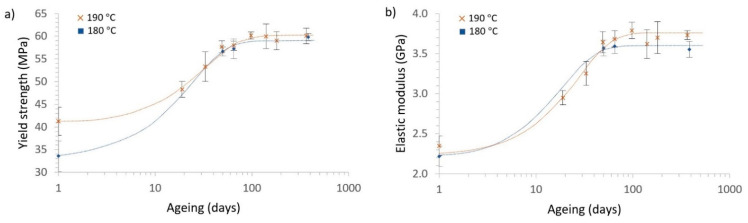
Evolution at different ageing times and printing temperatures (180 to 190 °C), with the corresponding fitting equations—logistic model–, for (**a**) the yield strength and (**b**) the elastic modulus.

Extruded at 190 °C:

(13) $$\sigma_{y}\left(\mathrm{MPa}\right)\;=\;\frac{59.9}{1\;+\;0.479e^{-0.0408t}}$$

(14) $$E\;\left(\mathrm{GPa}\right)\;=\;\frac{3.76}{1\;+\;0.715e^{-0.0541t}}$$

where $\sigma_{Y}$ is the yield strength in MPa, E is the elastic modulus in GPa, and t is the ageing time in days. Figure 10 shows Equations (11)–(14) fitted with the experimental data.

Contrary to what other authors have obtained, with the ageing technique used here, it was found that the values of the PLA properties increased [19]. This can be explained by the storing conditions used during the ageing in this study, for which we ensured low humidity conditions and protection from solar radiation.

Table 3 shows the parameters obtained from the logistic fitting. By using these parameters, we could compare the results more clearly. As shown in Figure 10, the ageing improved the mechanical properties of the PLA for both printing conditions, 180 °C and 190 °C, in a similar way.

Regarding $S_{\infty }$, the values for the yield strength and the elastic modulus in the steady state increased slightly (2–4%) with the printing temperature. These results align with those from previous studies in the literature, as printing at more elevated temperatures, below the degradation temperature, always provides materials with improved mechanical properties [71,72,73]. However, those studies researched printed structures, and the enhanced mechanical properties were related to the better adhesion between the printed material layers. Our results cannot be explained by this phenomenon, as we were working with a single filament, and another phenomenology has to be assessed.

**Figure 11:**
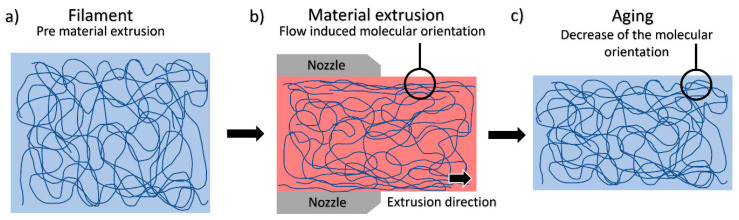
Schematised hypothesis of the flow-induced molecular orientation, relaxed during the ageing: (**a**) randomly distributed polymer chains; (**b**) extrusion process with the flow-induced molecular orientation on the surface; (**c**) ageing effect on the flow-induced molecular orientation; the orientation is partially lost.

To explain this improvement of $S_{\infty }$ with the printing temperature in single PLA filaments, the macromolecular dynamics of the PLA have to be addressed. The higher the printing temperature, the more likely it is that the polymer chains will align with the extrusion direction on the extruded material surface, a phenomenon called flow-induced molecular orientation. This produces compressive residual stress on the filament surface, something that occurs in more traditional processing methods like injection moulding [32,33,34]. This compressive state on the surface, attributable to the lower viscosity and better flow of the PLA during the extrusion, reduced the filaments’ superficial defects, which increased these mechanical properties for the higher printing temperatures studied. However, with ageing, this compressive stress resulting from the induced molecular orientation was partially lost due to the relaxation of the polymer chains, which were rearranged into less stressed configurations [74], as schematised in Figure 11. This can be explained for the extruded PLA by the fact that similar final properties were obtained for the steady state in the samples extruded at 180 and 190 °C, except for slightly higher values in the samples extruded at 190 °C, which was coherent with our results, considering the flow-induced crystallisation effect on the PLA at higher temperatures [75,76]. However, all the crystallinity values obtained were smaller than 0.5%, an expected result as the D-isomer content was higher than 2% in all our samples [75], and negligible differences were detected between samples.

Regarding the ageing rate, B, which was inversely related to the stabilisation time, it can be seen that the ageing rate was significantly reduced for both properties (up to 40%). This reduction indicated that it would take a longer time to reach a steady state. Let us consider the previous hypothesis that increasing the printing temperature increases the order in the polymer chains due to the flow-induced molecular orientation process. Logically, this leads to an increase in the difficulty of rearrangement and, therefore, a longer period of time to reach the steady state and a lower B, as more configurational chain rearrangements are required to reach the non-aligned stable configuration. This result is quite surprising because an increase of only 10 °C in printing temperature produced an abrupt reduction of around 40% in the ageing rate.

Finally, A’s ageing potential changed in a very different way for the elastic modulus and yield strength. There was a slight increase of about 4% for the elastic modulus and a reduction of 38% for the yield strength when increasing the printing temperature. It is not easy to find a relationship between this macroscopic behaviour and the micromechanisms operating in the material. However, if we go back to our previous hypothesis that an increase in the processing temperature induces both a higher alignment of the polymer chains and a reduction of surface defects in the extruded filaments, a significant increase in yield strength at t = 0 would result for the material extruded at a higher temperature. At the same time, the elastic modulus would remain almost unaltered for the different printing temperatures, as the presence of surface cracks does not influence this mechanical property, and no thermal degradation occurs at 190 °C in PLA [77]. As a consequence of the above points, as well as the evolution of $S_{\infty }$ mentioned previously, the temperature rise would significantly reduce the value of A for the yield strength due to the smaller probability of surface cracks forming during the extrusion, and a slight increase of A for the elastic modulus would be expected due to the flow-induced molecular orientation, as this phenomenon produces polymer chains well-aligned in the direction of the loads.

### 3.5. Microstructural and Fractographical Analysis

**Figure 12:**
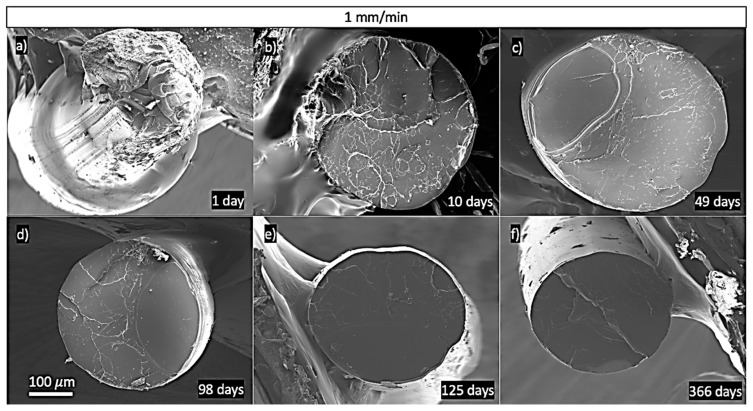
All samples tested at 1 mm/min ($\dot{\varepsilon }$ = 5 × 10^−1^ min^−1^) and extruded at 190 °C. Transversal view of PLA at: (**a**) 1 day of ageing, (**b**) 10 days of ageing, (**c**) 49 days of ageing, (**d**) 98 days of ageing, (**e**) 125 days of ageing, and (**f**) 366 days of ageing.

**Figure 13:**
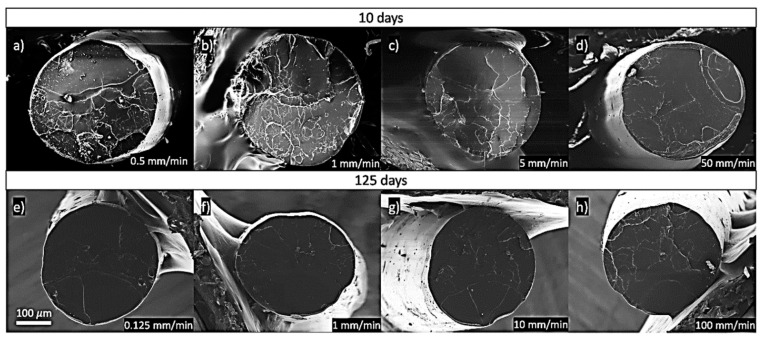
All samples extruded at 190 °C. Images (**a**–**d**): transversal view of PLA tested at 10 days of ageing and a strain rate of (**a**) 0.5 mm/min, (**b**) 1.0 mm/min**,** (**c**) 5 mm/min and (**d**) 50 mm/min. Images (e) to (h): transversal view of PLA tested at 125 days of ageing and a strain rate of (**e**) 0.1 mm/min, (**f**) 1.0 mm/min**,** (**g**) 10 mm/min and (**h**) 100 mm/min.

To understand the changes in the microstructural behaviour with ageing, the PLA’s fractographies are shown in Figure 12 and the strain rate in Figure 13. The plastic deformation was analysed by studying the surface roughness on each fracture surface.

Figure 12 shows the fractographies for the samples, extruded at 190 °C and tested at 1 mm/min, that were used for studying the effects of ageing on the mechanical properties. These provided shreds of evidence that increasing ageing produces more fragile PLA. These pieces of evidence concern the lower amount of plastic deformation in the more aged samples. The plastic deformation was assessed by observing the flatness of the surface roughness: the higher the flatness, the lower the plastic deformation. This reduction in the plastic deformation with age was coherent with our results, as we observed that the necking formation and cold drawing of the material happened less often with increased ageing. This phenomenon implies a massive plastic deformation of the material. Note how, in Figure 12a for one day of ageing, an image of the cold drawing on the filament indicates a high degree of plastic deformation. Comparing Figure 12d–f, it can be seen that, after 98 days of ageing, there were no differences on the fracture surfaces, which was in accordance with our hypothesis of a 100-day limit for the entire ageing of PLA.

Regarding these fractographies from Figure 12 and Figure 13, it can be seen the PLA behaviour was more brittle at higher strain rates and after longer periods of ageing. These facts were coherent with the experimental data, as we expected more brittle behaviour as the time given for the polymer chains to rearrange was reduced, and the flow of the polymer chains was reduced with ageing. This was indeed observed with the increasing enthalpic relaxation, indicating that the polymer chains had reached more stable locations inside the polymer. Moreover, no porosity was observed, which was consistent with the previously calculated porosity. Samples tested at 0.5 mm/min and aged for ten days tended to produce cold drawing. This phenomenon was less probable at 1 mm/min, and it was not observed at higher strain rates. Necking was not observed in samples tested at 0.125 mm/min and aged for 125 days, indicating lower chain mobility in the more aged specimens.

## 4. Conclusions

This study investigated the influence of the printing temperature, natural ageing, and crosshead speed on the mechanical, thermal, physical, and fractographical properties of PLA printed via material extrusion. The aim was to support the simulation of printed structures by starting from the properties of the individual filaments. This study further explored the understanding of the factors that affect 3D printed PLA filament properties, and the main conclusions are as follows:

1. Samples extruded at 180 and 190 °C were compared. It was noticed that the samples extruded at 180 °C had more morphological defects, such as wavy morphologies, due to the higher viscosity of the material. Therefore, the optimal printing temperature for PLA printed via material extrusion, with regard to its mechanical properties and morphology, is 190 °C.
2. The mechanical properties were highly dependent on the crosshead speed. The samples tested under low rates had smaller yield strengths and elastic moduli but higher deformations and plasticity, which was coherent with Eyring theory. Meanwhile, at faster speeds, the opposite happened. The 1 mm/min speed was chosen as a reference since it was the lowest speed that did not produce cold drawing. It helped in obtaining the minimum mechanical properties for the mechanical characterisation of the materials, as opposed to the faster speed, which was in the safe zone. In standard applications, the loading speeds for PLA can be expected to be much higher than 1 mm/min and thus the materials will exhibit superior mechanical properties during service. It is suggested to use the Eyring model to model the PLA’s behaviour with the crosshead speed.
3. The influence of ageing on the mechanical properties showed that the material should be aged for at least 100 days inside a PET zip-bag with desiccant and protected from direct solar radiation to reach the steady state.
4. Concerning the thermal properties, the materials were aged for up to 366 days for Tg, which stabilised at 100 days, similarly to the mechanical properties. However, the enthalpic relaxation can be expected to keep increasing even after 366 days. As expected from the theoretical point of view, negligible variations in the cold crystallisation and melting enthalpies were detected. The KWW model parameters for thermal properties were obtained and agreed with the values range for other polymers, and thus this model is suggested for modelling the thermal properties of PLA.
5. According to the mechanical testing results, a higher printing temperature (below the degradation temperature) can be expected to produce better flow-induced molecular orientation in the extruded filament, which provides higher mechanical properties. With ageing, these oriented chains and molecules slowly reach their stable configuration, but it takes more time to increase the printing temperature. These variations can be easily quantified by comparing the three parameters of the model for the different conditions of the material.
6. Microstructural and fractographical analysis of the filaments showed that the wavy morphologies in the filaments extruded at 180 °C did not affect the mechanical properties. The increases of the strain rate and ageing produced an essential change in the fracture morphology towards a flatter surface.
7. The influence of natural ageing on mechanical properties was fitted with a logistic model that predicts how the properties change with the ageing time in a low humidity atmosphere.

These results are crucial for future research since we used partial data to complete the material’s characterisation. Future work will aim to further research the fundamental properties of 3D printed PLA structures, such as the strength of a single PLA–PLA filament bond.

Temperatures of 200 °C and above were not investigated due to the limitations of the printing technique used to produce only one filament, rather than a scaffold, and ensure its round shape. Although it would have been interesting, as those temperatures are closer to the ones typically used for PLA, it is not experimentally possible for the moment. However, we provide essential information on which physical phenomena affect PLA during its processing via material extrusion or FDM. What is more, this information can be used to simulate the mechanical behaviour of the scaffolds and predict improved structures.

Lastly, the results provided in this article, together with additional measurements of PLA fibre-to-fibre bonding, could serve as a basis for modelling PLA and damages. In this way, optimal scaffolds for various applications could be optimised by employing computer simulations, without the need to print a multitude of structures and characterise them mechanically

## Figures and Tables

**Table 1:** Thermal properties of the extruded 1D PLA. All samples were extruded at 190 °C. T_g_: glass transition; T_ER_: enthalpic relaxation temperature; ΔH_ER_: enthalpy of enthalpic relaxation; T_CC_: cold crystallisation temperature; ΔH_CC_: enthalpy of cold crystallisation; T_m_: melting temperature; ΔH_m_: enthalpy of melting. Following IUPAC’s convention, positive enthalpy changes indicate that the material absorbs the energy, which is indicative of an endothermal reaction.

Ageing (Days)	T_g_ (°C)	T_ER_ (°C)	ΔH_ER_ (J/g)	T_CC_ (°C)	ΔH_CC_ (J/g)	T_m_ (°C)	ΔH_m_ (J/g)
1	51.7 ± 0.5	-	-	124 ± 1	−4.1 ± 0.2	151 ± 1	4.3 ± 0.3
3	53.1 ± 0.5	57.0 ± 0.5	0.9 ± 0.1	125 ± 1	−3.2 ± 0.1	150 ± 1	3.4 ± 0.2
7	55.2 ± 0.5	58.1 ± 0.5	1.4 ± 0.1	125 ± 1	−4.3 ± 0.2	151 ± 1	4.6 ± 0.3
14	57.4 ± 0.5	59.5 ± 0.5	2.0 ± 0.1	125 ± 1	−5.4 ± 0.4	151 ± 1	5.5 ± 0.4
21	58.3 ± 0.5	61.0 ± 0.5	2.5 ± 0.2	124 ± 1	−5.6 ± 0.4	151 ± 1	5.6 ± 0.4
28	58.8 ± 0.5	61.7 ± 0.5	2.8 ± 0.2	125 ± 1	−5.2 ± 0.4	152 ± 1	5.5 ± 0.4
42	59.2 ± 0.5	62.0 ± 0.5	3.2 ± 0.2	125 ± 1	−4.6 ± 0.3	151 ± 1	5.0 ± 0.3
84	59.9 ± 0.5	62.6 ± 0.5	3.8 ± 0.3	125 ± 1	−4.1 ± 0.3	151 ± 1	4.1 ± 0.3
104	59.9 ± 0.5	62.9 ± 0.5	4.0 ± 0.3	125 ± 1	−5.3 ± 0.4	152 ± 1	5.2 ± 0.4
125	60.0 ± 0.5	62.9 ± 0.5	4.1 ± 0.3	125 ± 1	−4.0 ± 0.3	150 ± 1	4.4 ± 0.3
140	59.9 ± 0.5	62.7 ± 0.5	4.2 ± 0.3	124 ± 1	−5.0 ± 0.3	151 ± 1	5.0 ± 0.3
240	60.1 ± 0.5	63.0 ± 0.5	4.4 ± 0.3	125 ± 1	−4.2 ± 0.3	151 ± 1	4.1 ± 0.3
366	60.0 ± 0.5	63.1 ± 0.5	4.5 ± 0.3	124 ± 1	−3.5 ± 0.2	150 ± 1	3.6 ± 0.2

**Table 2:** Mechanical properties of the PLA, followed by the root-mean-square error.

Printing Temperature (°C)	Wavy Surface	Ageing (Days)	Crosshead Speed (mm/min)	Strain Rate (min^−1^)	Yield Strength (MPa)	Elastic Modulus (GPa)
190	No	**10**	**0.5**	2.5 × 10^−2^	43 ± 1	3.0 ± 0.1
190	No	**10**	**1**	5 × 10^−2^	45 ± 2	3.2 ± 0.1
190	No	**10**	**5**	2.5 × 10^−1^	51 ± 3	3.5 ± 0.2
190	No	**10**	**50**	2.5	66 ± 2	4.1 ± 0.1
190	No	**125**	**0.125**	6.25 × 10^−3^	51 ± 1	3.2 ± 0.1
190	No	**125**	**1**	5 × 10^−2^	59 ± 1	3.5 ± 0.1
190	No	**125**	**10**	5 × 10^−1^	68 ± 3	3.5 ± 0.2
190	No	**125**	**100**	5	75 ± 2	3.9 ± 0.1
190	No	**426**	**0.125**	6.25 × 10^−3^	54 ± 1	3.1 ± 0.1
190	No	**426**	**1**	5 × 10^−2^	60 ± 2	3.6 ± 0.1
190	No	**426**	**10**	5 × 10^−1^	71 ± 1	3.6 ± 0.1
190	No	**426**	**100**	5	85 ± 2	3.9 ± 0.2
**180**	No	**1**	1	5 × 10^−2^	34 ± 3	2.2 ± 0.2
**180**	No	**49**	1	5 × 10^−2^	57 ± 1	3.5 ± 0.1
**180**	**Yes**	**49**	1	5 × 10^−2^	57 ± 2	3.5 ± 0.2
**180**	No	**65**	1	5 × 10^−2^	57 ± 2	3.6 ± 0.1
**180**	No	**384**	1	5 × 10^−2^	59 ± 1	3.6 ± 0.1
190	No	**1**	1	5 × 10^−2^	41 ± 3	2.3 ± 0.2
190	No	**19**	1	5 × 10^−2^	48 ± 2	2.9 ± 0.1
190	No	**33**	1	5 × 10^−2^	53 ± 3	3.3 ± 0.2
190	No	**49**	1	5 × 10^−2^	58 ± 1	3.6 ± 0.2
190	No	**65**	1	5 × 10^−2^	59 ± 1	3.7 ± 0.1
190	No	**98**	1	5 × 10^−2^	60 ± 1	3.8 ± 0.1
190	No	**140**	1	5 × 10^−2^	60 ± 2	3.6 ± 0.2
190	No	**180**	1	5 × 10^−2^	59 ± 2	3.7 ± 0.2
190	No	**366**	1	5 × 10^−2^	60 ± 2	3.7 ± 0.1

**Table 3:** Logistic fitting parameters and the relative variation (%) of each of them.

Parameter	Yield Strength		Relative Variation (%)	Elastic Modulus		Relative Variation (%)
T (°C)	180	190		180	190	
A (adim)	0.777	0.479	−38.3	0.678	0.715	5.46
$S_{\infty }$ (MPa)	58.8	59.9	1.87	3620	3760	3.87
B (days^−1^)	0.0631	0.0408	−35.3	0.0759	0.0541	−40.3
